# Web in the Neck – An Interesting Case Report

**DOI:** 10.1007/s12070-022-03434-1

**Published:** 2023-01-11

**Authors:** Meera N. Khadilkar, Sanchit Bajpai, Deviprasad Dosemane, Pooja K. Suresh

**Affiliations:** 1 Department of Otorhinolaryngology and Head & Neck Surgery, Kasturba Medical College, Mangalore, Manipal Academy of Higher Education, Manipal, Karnataka 575001, India; 2 Department of Pathology, Kasturba Medical College, Mangalore, Manipal Academy of Higher Education, Manipal, Karnataka 575001, India

**Keywords:** Branchial cyst, Head and neck neoplasms, Lymphangioma, Neck, Plexiform neurofibroma

## Abstract

Lateral neck masses are common in children, ranging from simple benign diseases to pathologies with malignant potential. Plexiform neurofibromas are extremely rare peripheral nerve sheath tumours involving multiple nerve sheath fascicles. They are typically seen in the paediatric population, with the majority affecting the craniofacial area and neck. Due to the close clinical and histological resemblance with other benign neck lesions such as lymphadenitis and branchial cysts, these cases can often go misdiagnosed. We describe a lesion in a young girl who presented with a progressive lateral neck swelling and how it was managed.

## Introduction

Lateral neck masses are quite commonly seen in children. Causes include infectious lymphadenitis, congenital lesions such as branchial cysts, cystic hygromas, thyroglossal duct cysts, dermoid cysts, neoplastic lesions such as leukaemia or lymphoma, and vascular lesions [1, 2]. A thorough history and clinical examination aid in narrowing down the diagnosis. This case report describes a long-standing lateral neck mass in a young girl, and how it was managed.

## Case Report

A 16-year-old female presented to the outpatient department with a diffuse swelling on the right side of the neck for five years; it was insidious in onset, with no aggravating or relieving factors. There was no pain or change in size of the swelling, and no symptoms suggestive of inflammation or compression of neck structures.

**Fig. 1:**
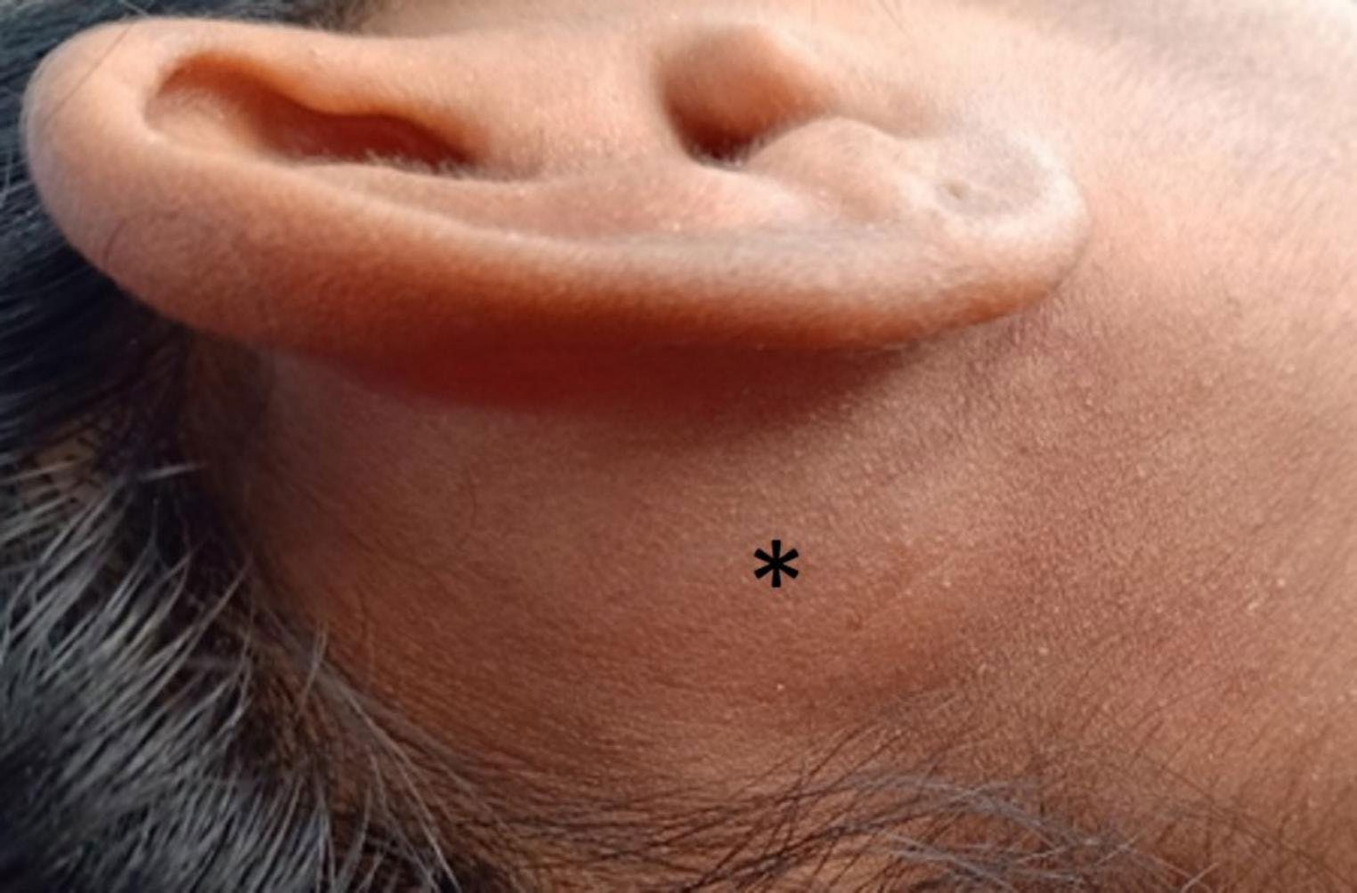
Clinical picture of lesion (black asterisk) on right side of neck

Local examination revealed an ill-defined swelling of size 10 × 5 cm on the right side of neck, which was soft, globular, cystic, non-tender, non-mobile, non-reducible, with lobulated margins. It extended from the body of mastoid, anteriorly and superiorly involving the cymba concha and right infra-auricular area, posteriorly up to the posterior border of sternocleidomastoid and inferiorly extending 2 cm above the level of mid-third of clavicle (Fig. 1). Skin above the swelling was normal and pinchable with no visible dilated veins or sinuses. Multiple lymph nodes were palpable apart from the swelling in right posterior triangle of neck and submandibular region; they were soft, tender, and mobile. The rest of ear nose throat examination was unremarkable.

**Fig. 2:**
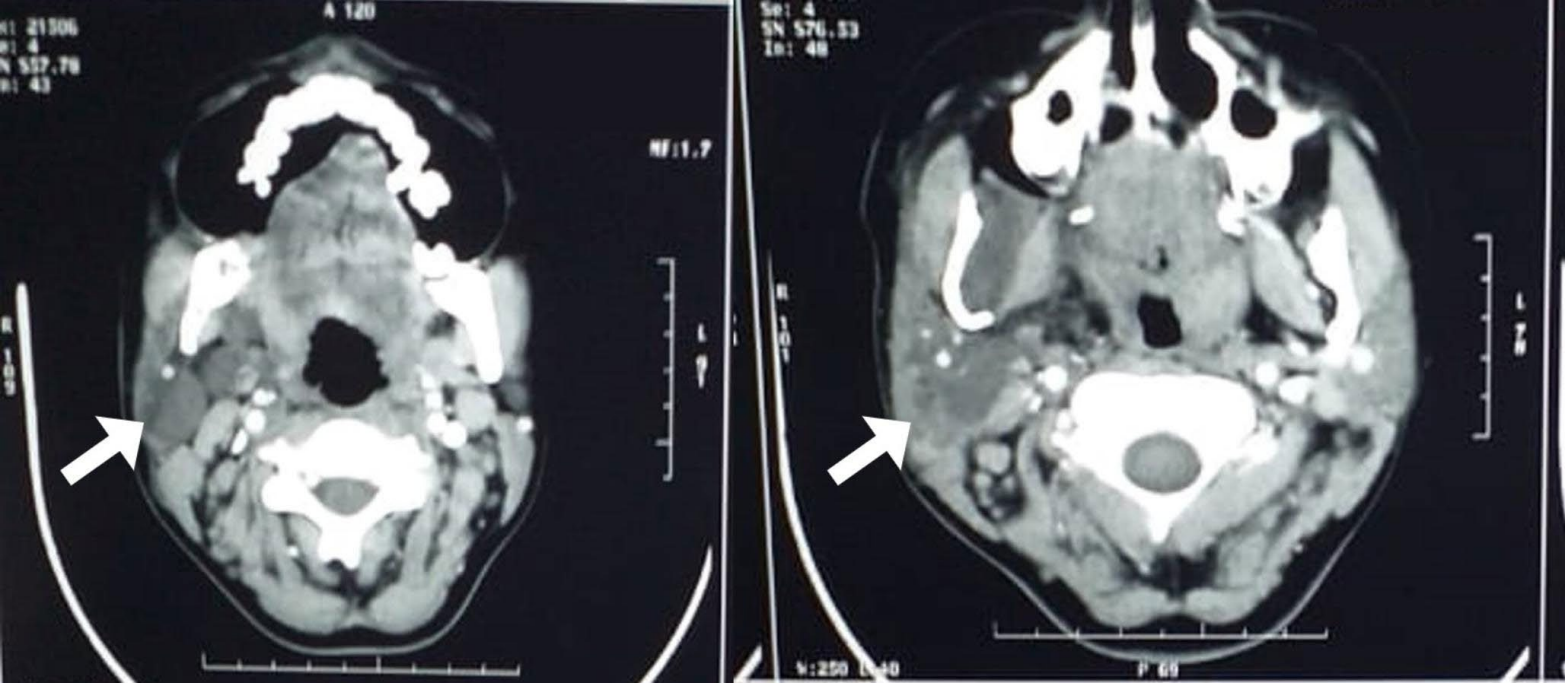
Contrast Enhanced Computed Tomography image of neck of lesion (white arrow)

Routine blood parameters were normal. Contrast Enhanced Computed Tomography (CECT) neck revealed an irregular, non-enhancing lobulated soft tissue lesion measuring 10.3 × 5.4 × 1.5 cm involving right parapharyngeal space, right masticator space involving the right medial and lateral pterygoid muscles, extending superiorly up to the mastoid body and a part of right parotid gland anteriorly, and inferiorly extending up till the level of mid-third of clavicle suggestive of lymphangioma (Fig. 2). Multiple nodes were also noted in right level V and level IB with the largest measuring 1.5 cm in right level IB. Fine needle aspiration of the lesion was inadequate for cytology opinion.

**Fig. 3:**
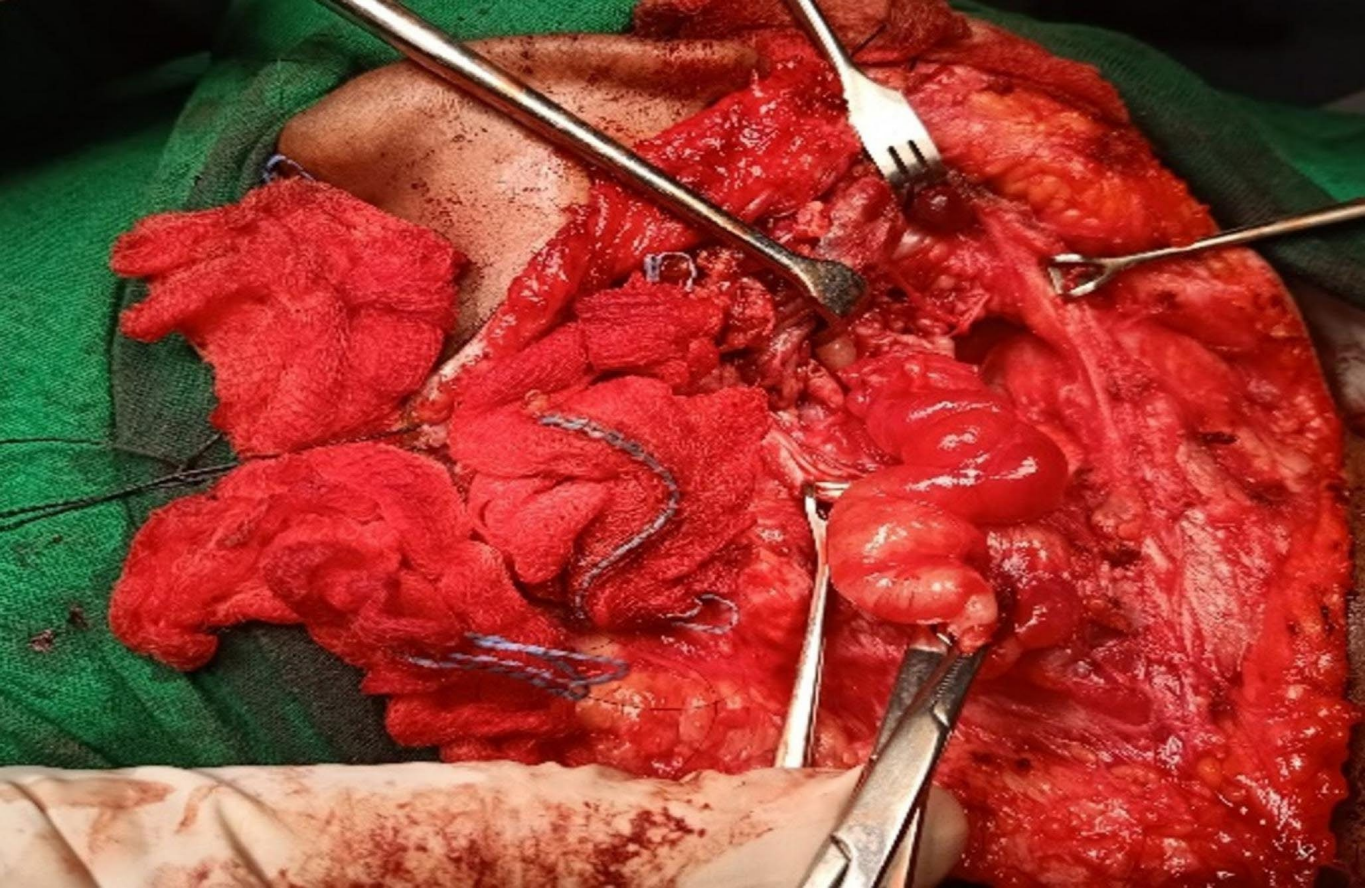
Intraoperative picture of excision of lesion

The patient thereafter underwent excision of the lesion under general anaesthesia. A hockey stick incision was made; soft tissue dissection was done. Multiple lesions were found adjacent to the carotid sheath and also in right parapharyngeal, retropharyngeal and submandibular spaces. They were cleared superiorly from the mastoid body, cymba concha, tail of parotid gland and submandibular space sparing the normal glands. Carotid sheath and its contents were preserved. The lesion was carefully separated using blunt dissection and bipolar diathermy. The tumour was completely excised in three parts, largest measuring 4 × 3 × 2 cm and smallest measuring 3 × 1.5 × 1 cm. The specimen was then sent for histopathological examination (Fig. 3). Postoperative period was uneventful; she was followed up for a year and was clinically asymptomatic.

**Fig. 4:**
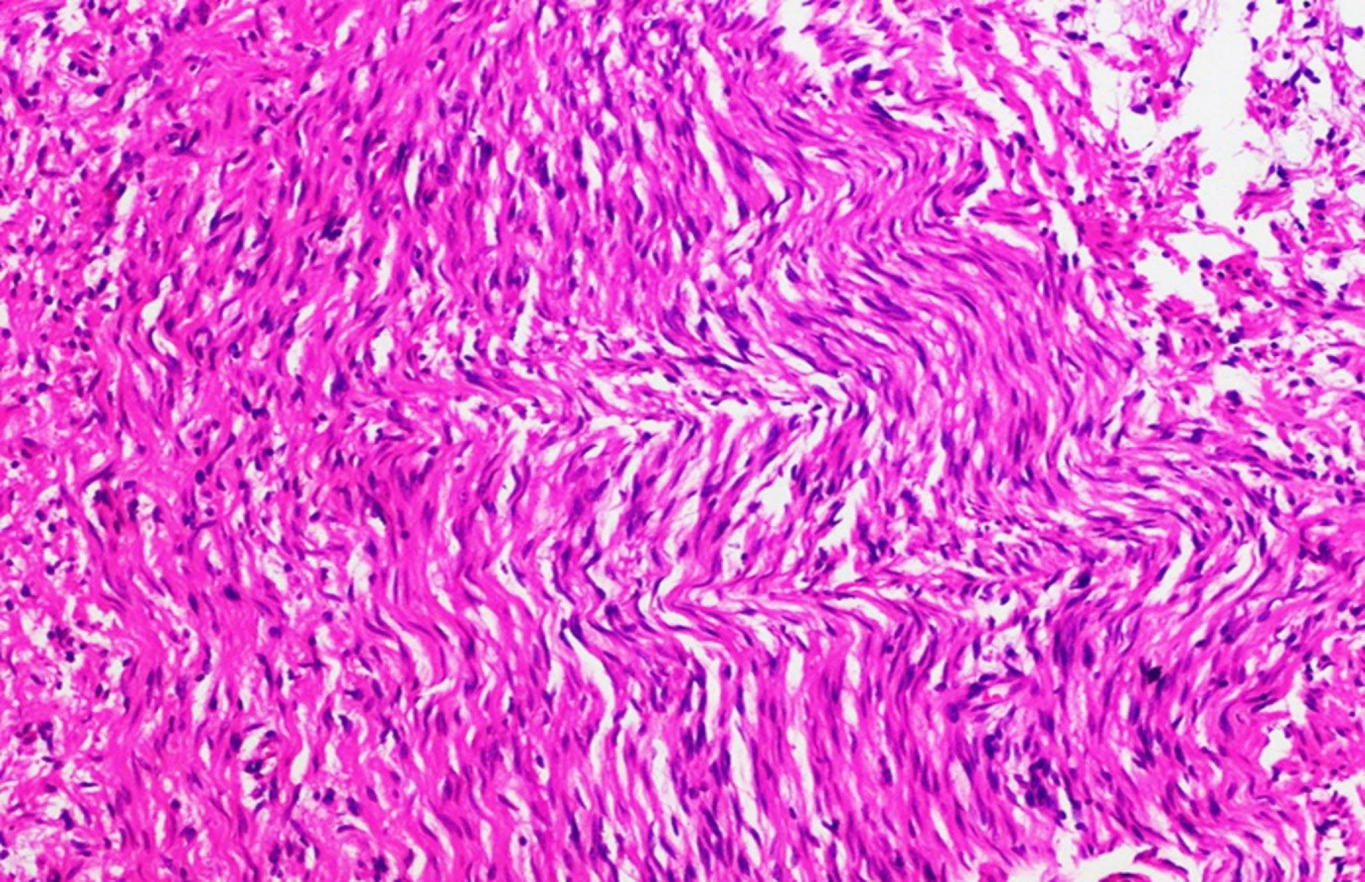
Histopathological image of lesion

The histopathological sections showed multiple nodules of expanded tortuous nerve bundles composed of spindle-shaped cells arranged in fascicles. The cells had an indistinct cytoplasmic border, spindle-shaped wavy nuclei with buckling, embedded in fibromyxoid stroma, strongly suggestive of plexiform neurofibroma (Fig. 4).

## Discussion

Based on the clinical features, differential diagnoses of lymphangioma and branchial cyst were considered in our patient. Lymphangiomas are common in the paediatric population and involve the posterior triangle of neck in 75% of cases, [3] whereas most of the branchial cysts (95%) arise from the second branchial arch and are typically situated along the anterior border of sternocleidomastoid as a single, painless neck mass, in second to fourth decade of life [4]. Radiologically, lymphangiomas appear hypoattenuated on CT; branchial cysts lie lateral and anterior to great vessels of the neck and may be adherent to internal jugular vein or even extend between external and internal carotid arteries [5]. Low attenuation of the lesion without significant distortion or compression of the vessels was noted in our patient.

Since cytology was inconclusive, we had no definite preoperative diagnosis; the presence of plexiform neurofibroma (PNF) on histomorphology was unexpected. PNF is a type of neurofibroma, a tumour of the peripheral nervous system, which is a hallmark of Neurofibromatosis (NF) type 1 (von Recklinghausen disease). NF-1 is a rare genetic disorder occurring in 1 per 3000 live births, with an autosomal dominant inheritance trait in 50% of the cases, and arises due to mutation of NF-1 tumour suppressor gene located on 17q22.1 chromosome which encodes a protein neurofibromin [2, 6, 7]. Various types of neurofibromas may develop based on the stage in which NF-1 mutation in Schwann cells occurs. PNF are congenital tumours, with a tendency to cause diffuse overgrowth of the area involved [6]. They occur in about 30% of cases of NF-1; isolated PNF is seldom seen. Positive family history is noted in half of the cases [8].

PNF is slow growing, well-bordered but unencapsulated, with intermittent tenderness and may involve adjacent tissues, leading to deformity, pain and morbidity. Cranial nerves, upper cervical nerves, scalp, skull base and postaural region may be involved in the head and neck [8] Gross morphology is described as a bag of worms due to its convoluted and complex shape [7]. The term “plexiform” refers to the network or web-like interlacing fascicles of elongated, wavy cells abundant in collagen on histopathology [9].

Based on National Institutes of Health consensus diagnostic criteria for NF -1, our patient had two features that confirmed the diagnosis [8]. She had a PNF proved on histopathology and a history of NF – 1 in her first-degree relative, which was elicited after obtaining the histopathology report. Once the clinical diagnosis is made, multidisciplinary team-based management comprising a physician, surgeon, neurologist, and geneticist is required. Genetic testing was not done on our patient due to financial constraints. It may also be beneficial in suspicious cases that do not meet the diagnostic criteria and in young patients with a serious tumour in whom early identification can affect treatment strategy [8]. Genetic counselling may be offered to assess family risk, family planning and the likelihood of Cowden syndrome when the diagnosis is uncertain [10]. Magnetic Resonance Imaging (MRI) is the imaging modality of choice for PNF, which will show high signal intensity in T2 weighted study, with a central area of low signal [8]. However, in our patient, CECT was performed. Not many reports of involvement of deep neck spaces were noted in recent literature. One case of PNF extending into parapharyngeal and posterior cervical spaces and mediastinum was reported in a two-year-old child [11].

Presently, surgery remains the treatment of choice for PNF; involvement of adjacent tissues, recurrence and large tumour burden worsens prognosis [8]. Separating PNF from normal nerve is challenging; complete excision may necessitate sacrifice of the nerve. Though acceptable in superficial lesions, deep lesions can be managed only after weighing the pros and cons of the neurological deficits of surgery and patient morbidity; nerve-related complications may occur in up to 10% of cases [9, 11]. Extensive lesions may lead to profuse intraoperative haemorrhage; hence early surgical intervention is advisable [2]. Radiotherapy is contraindicated in genetically predisposed individuals due to risk of malignant transformation. Chemotherapy has not been found to alter survival rate [8]. Interferon alpha has been reported recently as a useful adjunct in treating extensive and diffuse masses, especially in unresectable disease, and to reduce tumour volume and cranial nerve compression symptoms [12]. Paediatric age, head and neck involvement and incomplete resection are risk factors for tumour progression and recurrence [2].

Due to high risk of malignant transformation (up to 15%) [6] suggested by sudden onset of pain, increase in size, induration, and increased risk of recurrence (up to 20%) [2] our patient was kept on close follow-up for a year and is symptom-free till date.

## Conclusion

PNF must be considered as a diagnosis in lateral neck masses in paediatric patients, especially in those with a family history of NF-1. Early and complete surgical resection and regular review are essential to prevent malignant transformation and recurrence.

## Data Availability

Data transparent.
